# Acromegaly: The Relationship between Hemodynamic Profiles Assessed via Impedance Cardiography and Left Ventricular Systolic Function Assessed via Echocardiography

**DOI:** 10.3390/jcm13185630

**Published:** 2024-09-23

**Authors:** Agnieszka Włochacz, Paweł Krzesiński, Beata Uziębło-Życzkowska, Przemysław Witek, Grzegorz Zieliński, Anna Kazimierczak, Robert Wierzbowski, Małgorzata Banak, Grzegorz Gielerak

**Affiliations:** 1 Department of Cardiology and Internal Medicine, Military Institute of Medicine, National Research Institute, 01-982 Warsaw, Poland; 2 Department of Internal Medicine, Endocrinology, and Diabetes, Medical University of Warsaw, 02-106 Warsaw, Poland; 3 Department of Neurosurgery, Military Institute of Medicine, National Research Institute, 01-982 Warsaw, Poland

**Keywords:** acromegaly, echocardiography, impedance cardiography

## Abstract

**Background/Objectives**: Acromegaly-induced prolonged exposure to growth hormone and insulin-like growth factor 1 may have significant cardiovascular effects. The purpose of this study was to assess the relationship between hemodynamic parameters measured via impedance cardiography (ICG) and parameters of systolic left ventricular function measured via echocardiography in patients with acromegaly. **Methods**: The observational cohort study included 33 patients with newly diagnosed acromegaly, with a mean age of 47 years and without significant comorbidities. Correlation analysis (Spearman’s rank correlation coefficient R) was performed on parameters obtained by ICG and left ventricular systolic function parameters obtained by echocardiography. ICG assessment included indices of (1) cardiac function as a pump: stroke volume index (SI), cardiac index (CI), Heather index (HI), velocity index (VI), and acceleration index (ACI); (2) afterload: systemic vascular resistance index (SVRI) and total arterial compliance index (TACI); and (3) thoracic fluid content (TFC). Echocardiographic examinations evaluated left ventricular ejection fraction (LVEF) and global longitudinal strain (GLS). **Results**: A lower LVEF was associated with a lower SI (R = 0.38; *p* = 0.03) and a higher SVRI (R = −0.35; *p* = 0.046), whereas lower GLS was associated with lower SI (R = 0.43; *p* = 0.02), CI (R = 0.62; *p* < 0.001), VI (R = 0.59; *p* < 0.001), ACI (R = 0.38; *p* = 0.048), HI (R = 0.59; *p* < 0.001), and TACI (R = 0.50; *p* = 0.006) and a higher SVRI (R = −0.59; *p* < 0.001). No significant correlation was observed between either LVEF or GLS and TFC. **Conclusions**: In patients with acromegaly, poorer echocardiographic parameters of left ventricular systolic function are associated with impaired function of the heart as a pump and higher afterload as assessed via ICG.

## 1. Introduction

Acromegaly (AC), which involves prolonged exposure to growth hormone (GH) and insulin-like growth factor 1 (IGF-1), may have significant cardiovascular effects, including hemodynamic dysfunction of the heart and blood vessels [1,2,3]. Detecting subclinical cardiovascular dysfunction in patients with AC seems to be key in identifying increased cardiovascular risk and preventing cardiovascular complications, including symptomatic heart failure, which may develop at an advanced stage of the disease [4,5,6]. Therefore, accurate assessment of left ventricular (LV) systolic dysfunction is an important part of clinical examination and prognosis in patients with AC [7,8,9]. The LV ejection fraction (LVEF) assessed via conventional echocardiography is within the normal range in most patients at the time of AC diagnosis. However, a more precise assessment of myocardial strain via speckle-tracking echocardiography may already reveal subclinical LV systolic dysfunction in the early stages of AC [5,10,11]. Global longitudinal strain (GLS) assessments have proven more sensitive than conventional LVEF assessments in detecting impaired LV systolic function and early acromegalic cardiomyopathy in asymptomatic AC patients [5,10,11]. It has been demonstrated that impaired GLS is associated with an increased LV afterload and subsequent alterations in cardiovascular haemodynamics [12]. However, the complex mechanism of hemodynamic disorders, which involves GLS impairment in patients with AC, has not been elucidated.

Impedance cardiography (ICG) may provide valuable data on the interaction between the left ventricle and systemic arteries in patients with AC [13]. This modality has demonstrated its clinical significance as a method of assessing cardiac hemodynamic function, arterial compliance, and thoracic fluid content (TFC) in patients with heart failure, arterial hypertension (HTN), and coronary artery disease [14,15,16,17]. Preliminary data suggest ICG may be also useful in assessing patients newly diagnosed with active AC, as a means of detecting subclinical hemodynamic disorders in the early stages of AC [5,10,11].

In light of the above, we posited a hypothesis that a comprehensive hemodynamic assessment via ICG would help increase our understanding of the etiology and hemodynamic consequences of LV systolic dysfunction indicated by the echocardiographic parameters GLS and LVEF. Therefore, the objective of this study was to assess the correlation between hemodynamic parameters assessed via ICG and the echocardiographic parameters of LV systolic function in patients newly diagnosed with AC.

## 2. Materials and Methods

### 2.1. Study Design

Our observational cohort study carried out at the Military Institute of Medicine—National Research Institute, in 33 patients with early-stage AC who had not previously undergone endocrinological or neurosurgical treatment (18 males, mean age 47 years, 54.5% of patients with controlled HTN [mean blood pressure 121/77 mmHg]), involved thorough assessments of cardiovascular hemodynamic and echocardiographic parameters, cardiovascular risk factors, and symptoms indicating cardiovascular disorders. The study was designed and executed in compliance with the ethical standards set forth in the Declaration of Helsinki and Good Clinical Practice guidelines. The Ethics Committee of the Military Institute of Medicine—National Research Institute approved the study protocol (approval number 76/WIM/2016), and each subject signed a written informed consent form. In this paper, we present a secondary analysis of the study.

### 2.2. Clinical Examination and Blood Chemistry

The clinical examination included history of cardiovascular symptoms and cardiovascular risk assessment based on constitutional factors: body mass index (BMI), heart rate, and office systolic and diastolic blood pressure measured with an electronic blood pressure sphygmomanometer (Omron M4 Plus, Omron Corporation, Kyoto Head Office, Kyoto, Japan) in accordance with European Society of Cardiology (ESC) guidelines [18]. History taking included potential comorbidities, smoking status, and family history of cardiovascular disease. We reviewed patients’ records for history of dyslipidemia and carbohydrate metabolism disorders. Laboratory blood tests included a lipid profile, fasting blood glucose, and oral glucose tolerance testing (OGTT), as well as renal function tests (estimated glomerular filtration rate).

### 2.3. Study Population

Active acromegaly was diagnosed in accordance with current European Society of Endocrinology (ESE) guidelines, based on clinical manifestations, hormone levels, and imaging study results [19]. The patients included in this study had been newly diagnosed with active AC and had no clinically significant comorbidities nor history of either medical or neurosurgical pituitary tumor treatment. Clinical examination included the presence and duration of typical somatic manifestations of AC. Laboratory test result abnormalities considered typical of AC included GH and IGF-1 concentrations above the age- and sex-specific upper limits and no GH suppression two hours after glucose overload as part of 75 g OGTT (with immunohistochemically assessed GH levels of <46 pmol/L (1.0 μg/L). To exclude hypopituitarism and primary hyperthyroidism, patients underwent pituitary function assessments, which included adrenocorticotropic hormone (ACTH), thyroid-stimulating hormone (TSH), luteinizing hormone (LH), and follicle-stimulating hormone (FSH) levels. Moreover, all patients underwent magnetic resonance imaging of the central nervous system, which demonstrated a pituitary tumor in each case.

Study exclusion criteria were conditions adversely affecting LV hemodynamic function: (1) heart failure with midrange or reduced ejection fraction (LVEF < 50%); (2) clinically significant valvular disease; (3) coronary artery disease, including history of myocardial infarction; (4) history of pulmonary embolism; (5) central nervous system conditions, including documented history of a stroke or transient ischemic attack; (6) chronic kidney disease (estimated glomerular filtration rate < 60 mL/min/1.73 m^2^); (7) peripheral vascular disease; (8) severe chronic obstructive pulmonary disease (predicted Tiffeneau–Pinelli index <50%); (9) respiratory failure (PaO_2_ < 60 mmHg and/or PaCO_2_ > 45 mmHg); (10) previous head injury; (11) pregnancy; (12) lack of informed consent; (13) age < 18 years; (14) poor acoustic windows, defined as two or more myocardial segments inadequately visualized in echocardiography.

### 2.4. Echocardiographic Assessment

Once clinically significant comorbidities were excluded, each patient newly diagnosed with AC underwent a two-dimensional echocardiography examination involving the standard, American Society of Echocardiography-recommended parasternal, apical, and substernal views, obtained with a 4.6 MHz transducer (VIVID E 95 GE Medical System, Wauwatosa, WI, USA) [20].

All patients underwent longitudinal strain assessment, which was an assessment of longitudinal (subepicardial) myocardial fiber deformation directed from the base to the apex, via automated function imaging with EKG-gated images from the three basic apical views. Negative values represented fiber shortening, and positive values represented fiber lengthening or thickening. Longitudinal strain assessment helped obtain data on segmental and global LV function. We present the results in the form of a bullseye-type pie chart. We evaluated strain both globally and for the individual LV walls. The data from all three views are presented as mean GLS values. The normal range of GLS values is expressed as a proportion and is 20% [21].

### 2.5. Impedance Cardiography

All patients underwent ICG, employing a Niccomo device (Medis, Ilmenau, Germany) and a sphygmomanometer after a rest period of 10 min in the supine position. The data were obtained through the 10 min recording period and subsequently exported into a dedicated software program (Niccomo Software, 1.6 version). Based on the ICG recordings, we analyzed hemodynamic parameters including heart rate and the systolic and diastolic blood pressure. ICG assessments included (1) indices of cardiac function as a pump: cardiac output (CO [mL/min]), CI [mL*m^2^*min^1^]; stroke volume (SV [mL]); SV index (SI [mL/m^2^]); acceleration index (ACI [100*Z0*s^2^]: ACI = 100*dZmax*dt^1^), reflecting the maximum acceleration of the blood flowing out of the left ventricle; velocity index (VI [1000*Z0*s^1^]: VI = 1000*dZmax*Z0^1^), reflecting the maximum volume of blood flowing out of the left ventricle; Heather index (HI [Ohm*s^2^]): HI = dZmax*TRC), which characterizes the maximum contractility and reflects cardiac inotropism; (2) indices of afterload: systemic vascular resistance (SVR [dyn*s*cm^5^]; SVR index (SVRI [dyn*s*cm^5^*m^2^]); total arterial compliance index (TACI); and (3) TFC, which is a parameter of preload. For this study, we adopted a cutoff SI value of <35 mL/m^2^ and a cutoff TFC value of >35 1/kOhm for high cardiovascular risk [22,23,24].

### 2.6. Statistical Analysis

Digital filing and statistical analysis of data were performed using MS Office and Statistica 12.0 (StatSoft Inc., Tulsa, OK, USA). The results are presented as means ± standard deviation (SD), medians, and interquartile ranges for continuous variables and as absolute values (n) and proportions (%) for qualitative variables. The distribution of continuous variables was evaluated both visually and through the application of the Shapiro–Wilk test. The relationships between selected parameters of cardiovascular function or structure were analyzed with the Pearson correlation coefficient and the Spearman rank correlation coefficient, in accordance with the type of variable distribution. The level of statistical significance was set at *p* < 0.05.

## 3. Results

### 3.1. Baseline Characteristics

Demographic, clinical, and laboratory data of the study group are presented in Table 1.

The mean age of the 33 patients (including 18 males) was 47 ± 13 years, and the mean BMI was 27.8 kg/m^2^. Body weight in over 78% of patients was found to exceed normal limits, with 9 patients (27.3%) obese and 17 patients (51.5%) overweight. The mean blood pressure was 121/77 mmHg (with blood pressure < 140/90 mmHg in 94% of patients). Eighteen patients with AC (54.5%) had been diagnosed with HTN prior to this study. All these patients were receiving antihypertensive treatment, usually with one or two drugs; six patients (18.2%) were on beta-blockers, twelve (36.4%) were on angiotensin-converting enzyme inhibitors, one (3%) was on angiotensin receptor antagonists, and four (12.1%) were on diuretics.

The other most common comorbidities included dyslipidemia in three patients (9.1%), T2DM in six patients (18.2%), and prediabetes in ten patients (30.3%). The mean lipid levels were 189.6 mg/dL for total cholesterol, 92.7 mg/dL for triglycerides, 61 mg/dL for HDL cholesterol, and 121 mg/dL for LDL cholesterol. The mean serum creatinine was 0.76 ± 0.19 mg/dL.

Preserved function of the anterior pituitary was observed in 31 of the 33 patients. Eight patients with an invasive somatotropic tumor had been diagnosed with TSH deficiency. However, the condition was effectively managed with stable doses of L-thyroxine.

Detailed echocardiographic and hemodynamic data obtained in our study population of patients with AC are presented in Table 2 and in our previous papers [11,13].

### 3.2. The Relationship between Echocardiographic Parameters of LV Systolic Function and Hemodynamic Parameters Assessed with ICG

Lower LVEF values were associated with lower SI (R = 0.38; *p* = 0.03) and with higher SVRI (R = −0.35; *p* = 0.046); impaired GLS was associated with lower values of SI (R = 0.43; *p* = 0.02), CI (R = 0.62; *p* < 0.001), VI (R = 0.59; *p* < 0.001), ACI (0.38; *p* = 0.048), HI (0.59; *p* < 0.001), and TACI (R = 0.50; *p* = 0.006) and with a higher SVRI (R = −0.59; *p* < 0.001). There were no significant correlations between either LVEF or GLS and TFC (Table 3).

## 4. Discussion

Our study demonstrated a relationship between echocardiographic parameters of LV systolic function and both ICG-assessed parameters of cardiac function as a pump and increased afterload in patients newly diagnosed with AC, with this relationship more pronounced in the case of GLS than LVEF.

Recent years have seen a growing interest in the identification of subclinical cardiac dysfunction in patients with AC, to enable therapeutic intervention and cardiovascular risk reduction [3,5,10,11,25]. Long-term tissue exposure to excessive anabolic effect of both GH and IGF-1 may induce structural and functional changes in the myocardium via directly affecting myocyte growth and contractility and indirectly affecting neuroendocrine function and peripheral vascular resistance, contributing to pro-atherogenic processes and a generalized inflammatory reaction [26,27,28,29,30,31]. Histological examination of the hearts of patients with AC revealed cardiomyocyte hypertrophy and interstitial fibrosis, which gradually altered the myocardial structure [32]. Cardiomyocyte hypertrophy results in altered myocardial relaxation, whereas interstitial fibrosis leads to increased myocardial stiffness. As the condition progresses, myocardial remodelling involving concentric hypertrophy increases LV stiffness, which impairs LV compliance and increases LV filling pressure. The LVEF in patients with AC remains within the normal range for an extended period. Nevertheless, it is evident that the myocardium does not adapt to the increasing load without any effect on its function. In the initial stages of the process, the increased load on the left ventricle is compensated for by a reduction in the tension experienced by the ventricle during systole. The subendocardial location of longitudinal myocardial fibres renders them more susceptible to elevated intracardiac pressure and ischaemia than mid-wall fibres, which are responsible for LVEF and remain unaltered for a longer period of time. Such persistent adverse haemodynamic conditions increase the risk of progressive deterioration in LV function, which may subsequently lead to the development of cardiomyopathy secondary to endocrine dysfunction [33,34,35,36].

The current study population comprised patients with early-stage AC without clinically significant cardiovascular diseases that could affect circulatory system hemodynamics. In total, 20% of the study population had T2DM and 25% had HTN, which is consistent with the reports by other authors who observed no significant effect of either T2DM or HTN on impaired GLS in people with AC [10,11]. Conventional echocardiographic assessment showed all patients to have LVEF within the normal range.

Our study showed a relationship between lower indices of cardiac function as a pump and indices of increased LV afterload (higher SVRI, lower TACI), and a similar relationship with impaired GLS. Such relationships with GLS were decidedly stronger than those with LVEF, since the latter parameter may have been normal despite progressive longitudinal myocardial fiber dysfunction [35,36]. This is explained by the anatomy and pathophysiology of myocardial maladaptation to hemodynamic overload. Effective pumping function of the heart is possible due to the multilayered structure of the left ventricle. LV long-axis shortening during systole occurs as a result of the inner longitudinal and the outer oblique layer, whereas LV transverse shortening is due to the middle, circumferential layer. Moreover, the base and apex of the heart make opposing circular movements. Longitudinal fibers are more vulnerable to intraventricular pressure and to ischemia since they lie subendocardially, unlike the circumferential and radial fibers, which lie intramurally. The higher vulnerability of the subendocardial layer and the associated underlying dysfunction of longitudinal fibers along with the fact that they are less affected by preload can help early detection of their potential pathology [37,38]. It is probably for that reason that LV GLS imaging via speckle-tracking echocardiography in patients with AC has proved to be more sensitive than LVEF in identifying impaired LV systolic function [5,10,11]. Popielarz-Grygalewicz et al., who evaluated patients with AC and normal LVEF, showed the former group’s GLS to be significantly lower than in the study population (−16.6% vs. −20.7%, *p* < 0.01), and this finding was irrespective of potential comorbidities such as diabetes or HTN [10]. Our previous study also demonstrated that, despite their normal LVEF, patients newly diagnosed with AC exhibited lower GLS than that in the control group (18.1 vs. 19.4%, *p* = 0.023) [11]. These findings were opposite to those reported by Volschan et al. [39] and Gadelha et al. [40], who observed no significant differences in GLS in patients with AC in comparison with that in a healthy control population matched in terms of sex, age, and rates of HTN and diabetes. Unfortunately, we cannot explain the discrepancy in these study results without precise data on disease stage, study group homogeneity, and previous treatment.

It is worth noting that impaired LV systolic function observed in AC is associated with increased afterload, expressed by such ICG-measured parameters as increased systemic vascular resistance and decreased artery compliance, which is consistent with earlier reports in other patient populations [41,42,43]. The relationship between both SVRI and TAC on one hand and GLS on the other may be explained by increased end-diastolic LV wall strain potentially leading to increased myocardial fibrosis, with the process first affecting the subendocardial layer, which is the most susceptible to injury [37,38]. At the same time, there is no evidence of a significant effect of preload (TFC) on LV contractility. The information about cardiac function as a pump obtained via ICG (ACI, HI, VI, CI, and SI) is consistent with echocardiographic data and further elucidates the hemodynamic consequences of adverse effects of GH and IGF-1 on the myocardium, which have been presented above.

Our observations are consistent with those reported by other authors evaluating patients with heart failure and essential HTN in terms of the associations between hemodynamic indices assessed via ICG and parameters of LV function assessed via echocardiography [22,23,44,45,46]. Abnormal GLS values in patients with HTN were shown to be associated with lower CI and higher SVRI [44,45]. Parrott et al. reproted that changes in CI assessed via ICG were strongly correlated with changes in LVEF assessed echocardiographically [23]; whereas Ramirez et al. identified a significant correlation between reduced LV systolic function and decreased values of blood flow parameters, such as VI and ACI [46]. Moreover, other reports suggest that ICG may be useful in patients with HTN for assessing LV dysfunction, whose important predictors are CI (*p* = 0.005), left cardiac work index (LCWI; *p* = 0.008), and SVR (*p* = 0.048) [22].

### 4.1. Clinical Implications

The results of our study may contribute to a better understanding of the hemodynamic basis of LV dysfunction in patients newly diagnosed with AC. We identified vasoconstriction and decreased cardiac function as a pump as hemodynamic phenomena associated with echocardiographic parameters of LV dysfunction. Since hemodynamic changes may be corrected via appropriate treatment, we suspect that complex comprehensive assessments of individual hemodynamic profiles in patients with AC may help determine therapeutic targets, reverse LV remodeling, and improve cardiac function as a pump. Impedance cardiography, through the assessment of the haemodynamic function of the cardiovascular system, appears to have considerable clinical significance and may be useful in the early diagnosis of subclinical cardiac dysfunction in patients with AC. In this population, the early detection of hemodynamic abnormalities prior to the onset of circulatory symptoms may have significant clinical implications, as the rapid implementation of appropriate prophylactic treatment may prevent the development of cardiovascular symptoms, which have a markedly detrimental impact on the prognosis of patients with AC. The utilisation of non-invasive ICG for the purpose of cardiovascular risk stratification, with a view to enhancing prognosis, is of considerable importance in this cohort of patients. Furthermore, the treatment of hypertension concomitant with AC through a more comprehensive grasp of the pathophysiology of hemodynamic disorders via ICG will facilitate more exact therapeutic intervention, thus enabling the individualisation of therapy and monitoring of treatment effects.

### 4.2. Limitations

We realize that the relatively small study population is a limitation of our study. However, the small sample size is a result of a low incidence of GH-secreting pituitary neuroendocrine tumors. Due to its secondary nature, the analysis was not preceded by sample size calculations. Therefore, the absence of significant results in our analysis do not exclude real correlations in the population. Moreover, we excluded patients with clinically significant cardiovascular dysfunction or other clinically significant comorbidities, whose prevalence among people newly diagnosed with AC is relatively high. Another limitation of our study was the fact that we did not analyze radial or circumferential myocardial strain. Our study group comprised primarily young and middle-aged individuals with no evidence of myocardial ischemia; therefore, we did not conduct any investigations to exclude asymptomatic ischemic heart disease, such as an exercise stress test and/or coronary angiography. As a result, our results should not be indiscriminately extrapolated onto the entire population of patients with AC. While interpreting our results, it is important to consider the potential effects of antihypertensive treatments, although we would like to emphasize that HTN was well controlled in all affected patients. One strength of our study is the fact that we recruited patients who had received neither medical or neurosurgical pituitary tumor treatment, which was a way to exclude any potential effects of such treatment.

It should be noted that speckle-tracking echocardiography suffers from a number of limitations, such as intervendor variability and dependence on the operator’s experience, on good image quality, on frame rate (low frame rates are associated with the loss of speckles and accuracy), on loading conditions, and finally, on extrinsic mechanical factors such as the chest wall conformation [47,48,49,50].

## 5. Conclusions

In patients newly diagnosed with AC, impaired parameters of LV systolic function as assessed via echocardiography are related to impaired cardiac function as a pump and a higher afterload as assessed via ICG; these relationships are more pronounced for GLS than for LVEF. ICG is useful in diagnosing hemodynamic disorders in patients with AC.

## Figures and Tables

**Table 1:** Clinical data of patients with acromegaly.

Variable	Mean ± SD (Median; Interquartile Range) or n (%)
Patients with Acromegaly	
Demographics	
Age [years]	47.0 ± 13.5 (47.0; 38.0–61.0)
Male sex	18 (54.5)
BMI [kg/m^2^]	27.8 ± 4.1 (27.7; 25.3–30.1)
BMI ≥ 30 kg/m^2^	9 (27.3)
Heart rate [bpm]	75.6 ± 10.7 (77.0; 67.0–82.0)
SBP [mmHg]	121.0 ± 11.2 (123.0; 115.0–127.0)
DBP [mmHg]	77.0 ± 9.7 (77.0; 72.0–81.0)
Clinical Characteristics	
Hypertension	18 (54.5)
Prediabetes Diabetes mellitus	10 (33.3) 6 (18.2)
LVEF [%]	62.8
Pharmacotherapy	
RAAS blockers	13 (40.6)
ACEI	12 (36.4)
DIURETYK	4 (12.1)
ARB	1 (3)
BB	6 (18.2)
CCB	8 (24.2)
Laboratory Tests	
Creatinine [mg/dL]	0.76 ± 0.19 (0.8; 0.6–0.9)
eGFR > 60 mL/min/1.73 m^2^	33 (100)
TC [mg/dL]	189.6 ± 32 (193.5; 172–214)
TG [mg/dL]	92.7 ± 29.7 (92; 65–123.5)
HDL [mg/dL]	61 ± 13 (58; 49–70.5)
LDL [mg/dL]	121.1 ± 29.9 (121; 103.5–140)

Abbreviations: ACEI—angiotensin-converting-enzyme inhibitors, ARB—angiotensin receptor blockers, BB—beta-blockers, BMI—body mass index; CCB—calcium channel blockers, DBP—diastolic blood pressure; eGFR—glomerular filtration rate, HDL—high density lipoproteins, LDL—low-density lipoproteins, LVEF—left ventricular ejection fraction, RAAS blockers—renin-angiotensin-aldosterone system blockers, SBP—systolic blood pressure; TC—total cholesterol, TG—triglycerides.

**Table 2:** Selected hemodynamic parameters in patients with acromegaly.

Variable	Mean ± SD (Median; Interquartile Range) or n (%)
Impedance Cardiography	
Data Basic	
HR [bpm]	73.0 ± 10.6 (71.0; 65.0–80.0)
SBP [mmHg]	119.3 ± 10.1 (122.0; 115.0–124.0)
DBP [mmHg]	76.3 ± 9.2 (76.0; 72.0–80.0)
LV Pumping Function	
SI [mL/m^2^]	44.0 ± 9.4 (43.0; 37.0–51.0)
SI < 35 mL/m^2^	6 (18.2)
VI [1*1000^−1^*s^−1^] CI [mL*m^−2^*min^−1^]	42.3 ± 11.5 (45.0; 35.0–50.0) 3.2 ± 0.7 (3.2; 2.7–3.6)
ACI [1/100/s^2^]	65.0 ± 22.5 (64.0; 50.0–79.0)
HI [Ohm/s^2^]	8.47 ± 3.20 (8.00; 6.70–11.60)
Afterload	
SVRI [dyn*s*cm^−5^*m^2^]	2135 ± 578.3 (2004; 1761–2387)
TACI [mL*mmHg^−1^]	1.9 ± 0.7 (1.7; 1.6–2.1)
Hydration Status	
TFC [1/kOhm]	38.5 ± 6.9 (38.7; 33.8–42.4)
TFC > 35 1/kOhm	23 (69.7)

Abbreviations: ACI—acceleration index; CI—cardiac index; DBP—diastolic blood pressure; HI—Heather index; HR—heart rate; SBP—systolic blood pressure; SI—stroke index; SVRI—systemic vascular resistance index; TACI—total artery compliance index; TFC—thoracic fluid content; VI—velocity index.

**Table 3:** Relationship between hemodynamic parameters and echocardiographic parameters of left ventricular systolic function in acromegaly.

Correlations: R (*p*)				
Hemodynamic Parameters	Echocardiographic Parameters			
	LVEF [%]		GLS [%]	
	R	*p*-Value	R	*p*-Value
HR [bpm]	−0.07	0.69	0.19	0.29
SBP [mmHg]	−0.11	0.56	−0.20	0.27
DBP [mmHg]	−0.37	0.04	−0.29	0.11
MBP [mmHg]	−0.28	0.12	−0.23	0.21
PP [mmHg]	0.11	0.54	0.05	0.77
SI [mL/m^2^]	0.38	0.03	0.43	0.02
CI [mL*m^−2^*min^−1^]	0.28	0.12	0.62	<0.001
SVRI [dyn*s*cm^−5^*m^2^]	−0.35	0.046	−0.59	<0.001
TACI [mL/mmHg*m^2^]	0.22	0.25	0.50	0.007
VI [1*1000^−1^*s^−1^]	0.34	0.053	0.59	<0.001
ACI [1/100/s^2^]	0.27	0.14	0.36	0.048
HI [Ohm/s^2^]	0.19	0.28	0.59	<0.001
TFC [1/kOhm]	−0.06	0.74	−0.22	0.23

Abbreviations: ACI—acceleration index; CI—cardiac index; DBP—diastolic blood pressure; GLS—global longitudinal strain; HI—Heather index; HR—heart rate; LVEF—left ventricular ejection fraction, MBP—mean blood pressure; PP—pulse pressure; SBP—systolic blood pressure; SI—stroke volume index; SVRI—systemic vascular resistance index; TACI—total artery compliance index; TFC—thoracic fluid content; VI—velocity index.

## Data Availability

The data presented in this study are available upon request from the corresponding author. The data are not publicly available due to privacy or ethical restrictions.
